# WiseScaffolder: an algorithm for the semi-automatic scaffolding of Next Generation Sequencing data

**DOI:** 10.1186/s12859-015-0705-y

**Published:** 2015-09-03

**Authors:** Gregory K. Farrant, Mark Hoebeke, Frédéric Partensky, Gwendoline Andres, Erwan Corre, Laurence Garczarek

**Affiliations:** 1 Sorbonne Universités, UPMC Univ. Paris 06, UMR 7144, Station Biologique, CS 90074, 29688 Roscoff cedex, France; 2 CNRS, UMR 7144 Adaptation and Diversity in the Marine Environment, Oceanic Plankton Group, Marine Phototrophic Prokaryotes team, Place Georges Teissier, CS 90074, 29688 Roscoff cedex, France; 3 CNRS, FR 2424, ABiMS Platform, Station Biologique, Place Georges Teissier, CS 90074, 29688 Roscoff cedex, France

**Keywords:** Next-generation sequencing, Genome assembly, Genome finishing, Genome scaffolding, Marine *Synechococcus*

## Abstract

**Background:**

The sequencing depth provided by high-throughput sequencing technologies has allowed a rise in the number of *de novo* sequenced genomes that could potentially be closed without further sequencing. However, genome scaffolding and closure require costly human supervision that often results in genomes being published as drafts. A number of automatic scaffolders were recently released, which improved the global quality of genomes published in the last few years. Yet, none of them reach the efficiency of manual scaffolding.

**Results:**

Here, we present an innovative semi-automatic scaffolder that additionally helps with chimerae resolution and generates valuable contig maps and outputs for manual improvement of the automatic scaffolding. This software was tested on the newly sequenced marine cyanobacterium *Synechococcus* sp. WH8103 as well as two reference datasets used in previous studies, *Rhodobacter sphaeroides* and *Homo sapiens* chromosome 14 (http://gage.cbcb.umd.edu/). The quality of resulting scaffolds was compared to that of three other stand-alone scaffolders: SSPACE, SOPRA and SCARPA. For all three model organisms, WiseScaffolder produced better results than other scaffolders in terms of contiguity statistics (number of genome fragments, N50, LG50, etc.) and, in the case of WH8103, the reliability of the scaffolds was confirmed by whole genome alignment against a closely related reference genome. We also propose an efficient computer-assisted strategy for manual improvement of the scaffolding, using outputs generated by WiseScaffolder, as well as for genome finishing that in our hands led to the circularization of the WH8103 genome.

**Conclusion:**

Altogether, WiseScaffolder proved more efficient than three other scaffolders for both prokaryotic and eukaryotic genomes and is thus likely applicable to most genome projects. The scaffolding pipeline described here should be of particular interest to biologists wishing to take advantage of the high added value of complete genomes.

**Electronic supplementary material:**

The online version of this article (doi:10.1186/s12859-015-0705-y) contains supplementary material, which is available to authorized users.

## Background

The drastic reduction of cost associated with Next Generation Sequencing (NGS) technologies has revolutionized the field of genomics, granting researchers an easy access to new genomes [1]. However, while complete genomes were a gold standard until the end of the 2000’s, the recent exponential increase of the number of sequenced genomes came along with a dramatic rise in the proportion of draft genomes since 2010 ([2]; see also *http://www.genomesonline.org/statistics*). Draft genomes may be sufficient for some applications, such as population genomics or multi-locus analyses. Still, the availability of complete genomes remains highly valuable when all the genes of an organism need to be properly identified. This is for instance the case in comparative genomic studies, where it is necessary to determine the pan, core and accessory genomes, or in the identification of genomic islands, which may be involved in pathogenicity and/or adaptation to specific ecological niches. In this context, bioinformaticians constantly have to adapt algorithms and pipelines to the rapidly evolving sequencing technologies, which generate genomic data with ever-increasing read length and sequencing depth, in order to successfully address issues raised by genome assembly and scaffolding.

Most recent *de novo* assemblers, such as VELVET [3, 4] or CLC AssemblyCell^©^ (CLC Bio, Aarhus, Denmark) are able to use paired reads, with short or long inserts (*i.e.,* pair-ends or mate-pairs, hereafter PE and MP, respectively) to assemble reads into contigs containing gaps filled with a number of Ns that is based on the average insert size of the PE and/or MP libraries. Although the use of paired reads generally improves the quality of the assembly, the simultaneous use of the sequences and insert sizes information shows some limitations for both library types. Indeed, although PE libraries allow one to get longer reads, and therefore improve local assembly, they do not permit to connect regions a few thousands nucleotides apart. While MPs allow to do so, generation of such long-insert libraries remains technically challenging, often resulting in large insert size variability [5–7], leading to an approximate number of Ns within contigs.

An alternative solution to the concomitant treatment of local and distant assembly is to perform consecutively the assembly into contigs, using single or PE reads, then scaffolding, *i.e.* ordering and merging contigs into scaffolds, using paired reads. The latter step is addressed by several stand-alone scaffolders such as Bambus [8], SSPACE [9], SOPRA [10], MIP Scaffolder [11] and, more recently, SCARPA [12]. The strategy adopted by the greedy algorithms Bambus and SSPACE consists in ordering contigs based on the number of links that associates them, while other scaffolders use more sophisticated algorithms to explore a graph where nodes and edges are contigs and pairs of reads linking them, respectively. Although most of these scaffolders are able to automatically produce a manageable number of scaffolds, none of them is able to generate a single scaffold. Indeed, numerous genomic features, essentially related to repeats (*e.g.*, multi-copy genes, homopolymers, etc.), are either difficult to reconstruct *in silico* or simply difficult to sequence, resulting in either gaps, chimerae or collapsed repeats in the genomic sequence that may lead to erroneous decisions during the manual finishing. Moreover, most stand-alone scaffolders are tricky to install and/or run and can require significant work to prepare input files [13]. At last, although scaffolding lowers the number of chromosome (Chr.) fragments, it does not avoid the need for genome finishing, a step during which gaps created through scaffolding are closed and local polymorphism associated with multi-copy genes is resolved. Altogether, these time-consuming steps might be discouraging and often result into genomes being published as drafts [2].

**Fig. 1:**
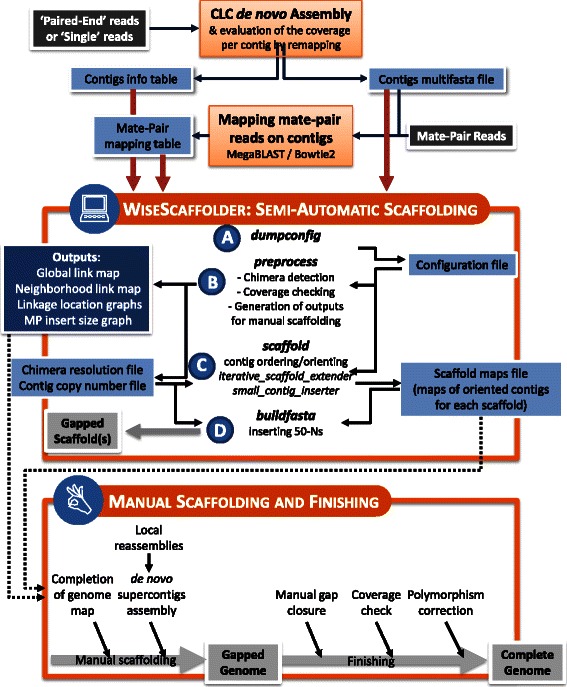
Flowchart describing the whole pipeline from genome assembly to finishing that includes the generation of input data for WiseScaffolder (top), the semi-automatic scaffolder itself (middle) and the manual scaffolding and genome finishing steps (bottom). Dark grey boxes correspond to raw sequencing data, light blue boxes to either input files for WiseScaffolder or to exchange files used during the scaffolding process, the dark blue box to the outputs of WiseScaffolder and light grey boxes to the different genome stages along the whole pipeline. The four capital letters correspond to the four subcommands of WiseScaffolders

In the context of a large marine *Synechococcus* genome sequencing project (*http://application.sb-roscoff.fr/cyanorak/*), we developed WiseScaffolder, an innovative bioinformatic tool that mimicks progressive manual scaffolding using a greedy, iterative reconstruction of contigs neighborhood (Fig. 1). Based on a simple set of rules, WiseScaffolder efficiently addresses such scaffolding issues as chimerae and multi-copy contigs. Besides semi-automatically generating high quality scaffolds, it also provides valuable data to manually improve the scaffolding and also useful for genome finishing. Here, the efficiency of WiseScaffolder was tested on one of the newly sequenced genomes of marine cyanobacteria, *Synechococcus* sp. WH8103 (hereafter WH8103), as well as on two additional datasets from the GAGE study (Genome Assembly Gold-Standard Evaluation; [14]), *Rhodobacter sphaeroides* and *Homo sapiens* Chr.14, representatives of the prokaryotic and eukaryotic domains, respectively. Quality of the results were compared to those obtained with three other stand-alone scaffolders (SCARPA, SOPRA and SSPACE) using QUAST [15] and other comparative genomic tools.

## Results and discussion

### Comparison of WiseScaffolder with other scaffolders

The recent advent of NGS technologies has triggered the development of a number of stand-alone scaffolders, the relative efficiency of which may be tricky to assess for bioanalysts and is highly dataset dependent. For instance, the *E. coli* strain K-12 substrain MG1655 dataset that was used to compare the recently published SCARPA to several other scaffolders [12], might be misleading because the assembly of such low quality data (insert size ~200 bp, reads ~36 bp) generally results into short contigs that are challenging to scaffold, whatever the efficiency of the scaffolder. Here, we rather chose to compare recent and/or widely used scaffolders using three genome datasets that were assembled from reads sequenced by Illumina with up-to-date performance (i.e., 101 bp paired reads, Table 1). These include the newly sequenced *Synechococcus* sp. WH8103 genome (42 contigs assembled using the CLC assembler; MP library insert size ~4 kb) as well as two reference datasets retrieved from the GAGE study [14], namely *R. sphaeroides* (177 Bambus2-assembled contigs [16]; MP library insert size ~3.5 kb) and *Homo sapiens* Chr.14 (3,541 CABOG-assembled contigs [17]; MP library insert size: 2.3-2.8 kb).

**Table 1:** Description of the three datasets used in this study

		*Synechococcus* sp. WH8103	*Rhodobacter sphaeroides*	*Homo sapiens* Chr.14
Genome	Genome size	2,429,688	4,603,060	88,289,540
	GC %	59.48	68.79	40.89
	Genome composition	1 x chromosome	2 x chromosomes	1 x chromosome
			5 x plasmids	
Pair-End library	Read length (bp)	101	n.a.	n.a.
	Nb of reads (*x*2)	2.0 × 10^7^		
	Average insert size (bp)	75		
Mate-Pair library	Read length	101	101	101
	Nb of reads	4.1 × 10^7^	2,05 × 10^6^	3.65 × 10^7^
	Maximal insert size	3,800	3,000	3,500
Contigs	Contigs assembler	CLC AssemblyCell©	Bambus2 [16]	CABOG [17]
	Nb of contigs	42	177	3541
	Nb of bp in contigs	2,398,638	4,371,571	86,255,201
	Reference	This study	[14]	[14]

*n.a.* not applicable

**Fig. 2:**
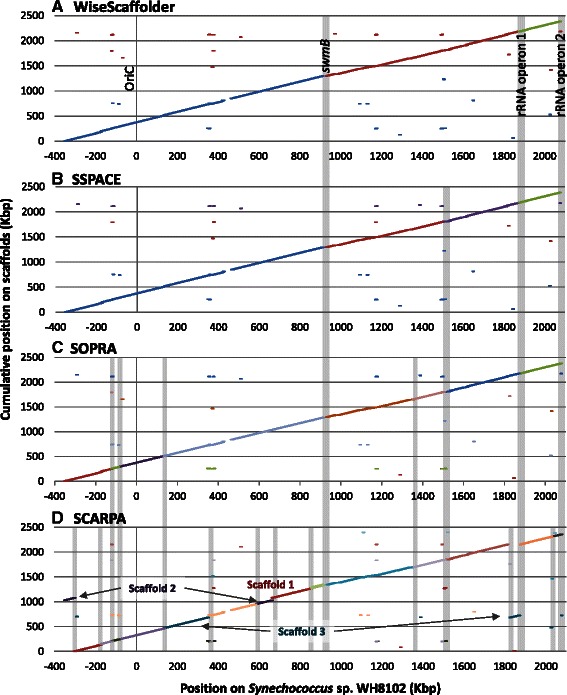
Comparison of the scaffolds of *Synechococcus* sp. WH8103 obtained using (**a**) WiseScaffolder, (**b**) SSPACE, (**c**) SOPRA and (**d**) SCARPA against the closely related genome, *Synechococcus* sp. WH8102. Whole genome alignments were realized using MUMmer [34]. Only scaffolds and contigs of *Synechococcus* sp. WH8103 larger than 10 kb are displayed and have been organized to be syntenic with the WH8102 genome. Breakpoints in chromosome reconstruction are represented by light grey areas. Note that the large gap around position 450 Kbp in all 4 assemblies corresponds to a genomic island with similar size but different gene content in *Synechococcus* spp. WH8102 and WH8103 (see also Additional file 1: Figure S1)

**Fig. 3:**
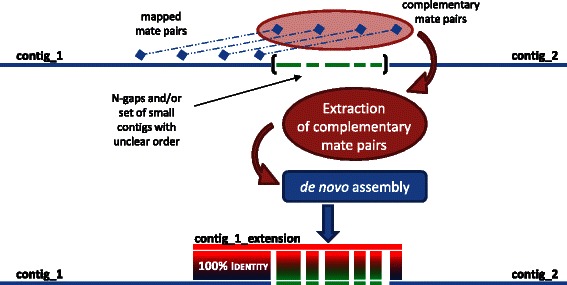
Local assembly at the edge of a contig using mate-pair reads. The extraction and local assembly of the mate sequences of reads mapping in the vicinity of a region of interest (in the present case, 3′-end of contig_1) allows its extension till the 5′-end of the neighboring contig_2

To assess the efficiency of WiseScaffolder, the abovementioned datasets were scaffolded using three other stand-alone scaffolders: SSPACE [9], SOPRA [10] and SCARPA [12]. Results of this benchmark are shown in Table 2. Additionally, for WH8103, we also tested the simultaneous assembly and scaffolding functions of the CLC assembler by assembling MP reads with distance constraint. In the case of the cyanobacterial genome, CLC assembly of MPs proved much less efficient in terms of scaffolds number and size than the stand-alone scaffolders and was thus not further considered in the benchmarking. For all datasets, WiseScaffolder produced better results than the three other scaffolders in terms of contiguity statistics, as attested by a generally smaller number of genome fragments (*i.e.*, scaffolds + unscaffolded contigs), the larger size of these fragments, as well as better contiguity indexes (N50, LG50 and LG75, Table 2). For instance, for both prokaryotes, WiseScaffolder was able to build a single scaffold covering more than 50 % of the whole genome (LG50 = 1), while for the *H. sapiens* Chr.14, this percentage was reached for 38 contigs with WiseScaffolder *vs.* 52, 74 and 460 for SCARPA, SSPACE and SOPRA, respectively. It is also noteworthy that SSPACE was also quite efficient on WH8103, while SCARPA proved the second best after WiseScaffolder for *R. sphaeroides* and *H. sapiens* Chr.14, according to contiguity indexes. Additional estimators provided by QUAST in contrast suggest that WiseScaffolder would result in more misassemblies than the other scaffolders. However, these estimators somewhat penalize WiseScaffolder because its purpose is not to assemble contigs at the sequence level like the other scaffolders. Instead, it generates an orientated map of contigs, in which the position and orientation of large contigs is unambiguous, but that of the small contigs in-between may be inaccurate due to the variability of MP insert size or the occurrence of collapsed repeats. This scaffolding strategy is highly secure, since the inter-contig regions comprising these small contigs (usually arisen from assembly bottlenecks) are only resolved after automatic scaffolding using contig extensions (Figs. 1 and 3), but it has for side effect to be interpreted by QUAST as relocation misassemblies (Table 2). Eventually, the global quality of the scaffolds produced by WiseScaffolder was also validated by the remarkable synteny of these scaffolds with that of the high quality reference genome of its close relative WH8102 (Figs. 2 and Additional file 1: Figure S1), with only three scaffolds covering most of the chromosome. By comparison, SSPACE and SOPRA performed almost as well in terms of synteny but with 1 and 5 additional scaffolds > 10 kbp, respectively, while SCARPA produced the most fragmented chromosome with only three scaffolds generated larger than 10 kbp, corresponding to only one fourth of the WH8102 genome. It must be noted that two of the three breakpoints common to all four assemblies correspond to the two identical copies of the ribosomal operon, whose size (~5.3 kb) exceeds the insert size of the MP library (~4 kb), whereas the third one corresponded to *swmB*, a gene coding for a giant protein (around 32 kb) involved in cell motility [18] that contains highly repetitive regions preventing a proper automatic assembly and scaffolding. The additional breakpoint observed in the SSPACE assembly compared to WiseScaffolder corresponds to two highly conserved hypothetical genes (orthologs of SYNW1563 and SYNW1565 in WH8102), both present in multiple copies in the two genomes. Many additional gaps were present in the assembly made with SCARPA and the absence of synteny of two of the three scaffolds with the WH8102 reference genome indicates the occurrence of chimerae (see Fig. 2d). Altogether, WiseScaffolder gave the best results on this dataset (Table 2 and Fig. 2) and allowed us to obtain a genome of much better quality than most currently published draft genomes. Furthermore, a key asset of WiseScaffolder compared to other scaffolders is that the ‘*preprocess’* subcommand also provides very useful outputs for resolution of chimerae (Fig. 1) that, together with multiple copy areas, often constitute scaffolding bottlenecks. These outputs can then be used for further improvement of the quality of the genome assembly.

**Table 2:** Comparative statistics for the assembly and scaffolding of *Synechococcus* sp. WH8103, *Rhodobacter sphaeroides* and *Homo sapiens* Chr.14

		*Synechococcus* sp. WH8103						*Rhodobacter sphaeroides*					*Homo sapiens* Chr.14				
		CONTIGS	SCAFFOLDS					CONTIGS	SCAFFOLDS				CONTIGS	SCAFFOLDS			
Contiguity Statistics	Assembler/Scaffolder	CLC	WISCA	SSPACE	SOPRA	SCARPA	CLC	Bambus2	WISCA	SSPACE	SOPRA	SCARPA	CABOG	WISCA	SSPACE	SOPRA	SCARPA
	Number of scaffolds	n.a.	3	13	5	7	36	n.a.	17	116	18	30	n.a.	228	930	414	259
	Unscaffolded contigs	42	13	n.a.	22	23	n.a.	177	33	n.a.	136	39	3,541	184	n.a.	2,587	468
	Fragments^a^ ≥ 10 kbp	17	3	4	8	15	13	72	16	59	64	34	2,132	221	435	1,885	362
	Max. scaffold size (kbp)	357	1,296	889	779	357	500	279	2,502	279	407	1,346	297	2,554	1,592	497	2,253
	N50^b^ (kbp)	211	1,296	729	367	222	278	97	2,502	134	118	178	47	694	348	56	499
	LG50^c^	5	1	2	3	5	4	17	1	13	14	5	563	38	74	460	52
	LG75^c^	8	2	3	5	8	6	38	5	27	31	13	1,217	79	161	1,008	115
	Nb of Ns (kbp)	n.a.	2.4	10.2	7.8	15.0	20.0	n.a.	8.9	35.0	11.0	55.6	n.a.	178.7	359.3	38.0	304.8
	Genome coverage (%)^d^	98.72	98.86	98.92	98.72	98.72	98.80	94.97	94.97	95.06	94.97	94.77	97.70	97.73	97.69	97.70	97.35
Misassemblies^h^	Misassemblies	0	13	2	2	5	2	4	62	7	6	10	108	981	177	120	200
	-Relocations^e^	0	11	2	2	5	2	1	48	3	2	6	106	743	175	118	199
	-Translocations^f^	0	0	0	0	0	0	3	8	4	4	4	0	0	0	0	0
	-Inversions^g^	0	2	0	0	0	0	0	6	0	0	0	2	238	2	2	1
	Misassembled contigs	0	2	1	1	3	2	4	10	6	6	7	87	177	105	96	101
	Local misassemblies	11	28	36	22	12	19	308	365	352	324	369	454	1,932	2,125	668	1,368
	Mismatches	2	7	20	2	7	34	254	287	279	268	280	87,135	86,626	86,856	87,335	85,748
	Indels	0	3	1	3	1	3	255	277	273	267	270	19,990	20,656	20,740	20,511	21,102
	-short indels	0	1	0	1	1	2	195	204	206	202	203	17,000	16,912	17,126	17,190	17,333
	-long indels	0	2	1	2	0	1	60	73	67	65	67	2,990	3,744	3,614	3,321	3,769
	Indels length	0	19	15	55	3	38	2,035	2,423	2,223	2,124	2,230	72,044	103,854	92,992	80,016	92,442

*n.a.* not applicable, *WISCA* WiseScaffolder, ^a^i.e. scaffolds and unscaffolded contigs; ^b^contig size over which the sum of contig sizes corresponds to 50 % of the assembly; ^c^minimal number of fragments (contigs/scaffold) to cover 50 %/75 % of the reference genome; ^d^calculated as $\frac{\varSigma \kern0.5em  contigs\kern0.5em  length-\kern0.5em Nb\kern0.5em  of\kern0.5em Ns}{\operatorname{Reference}\kern0.5em  genome\kern0.5em  length}\times 100$; ^e^misassembly where contiguous sequence fragments align on the same chromosome but where the left sequence fragment aligns over 1 kbp away from the right sequence fragment on the reference or overlap by more than 1 kbp; ^f^misassembly where the sequence fragments align on different chromosomes; ^g^misassembly where the sequence fragments align on opposite strands of the same chromosome; ^h^misassembly statistics are results from QUAST [15]

### Semi-automatic improvement of the scaffolding and finishing of the *Synechococcus* sp. WH8103 genome

Post-scaffolding improvements of the genome assembly are generally achieved by ordering and merging scaffolds into a reduced set of gapped sequences and then closing the remaining gaps either by wet laboratory PCR amplification, followed by Sanger sequencing, or by computational approaches. For instance, tools such as ABACAS can be used to organize contig/scaffolds in a syntenic order with respect to a reference genome [19], then IMAGE or GapFiller to tentatively close gaps using paired reads [20, 21]. However, both approaches have limitations. Indeed, while wet laboratory experiments are generally costly and time consuming [22], most current computational algorithms despite being faster, only partially address the scaffolding and finishing issues.

Here, we describe a complete workflow for semi-automatic genome finishing dealing with most frequently encountered difficulties, such as closing remaining gaps, resolving collapsed repeats and correcting sequences of highly conserved multi-copy genes. For this purpose, the maps of oriented contigs, generated using the *'scaffold'* sub-command, constitute a valuable tool for the manual scaffolding step (Fig. 1), by allowing one to easily manipulate contigs without immediately having to deal with their sequence information. In the case of WH8103, the manual improvement of this map using WiseScaffolder outputs allowed us to obtain a circular map of the complete genome without having to use a reference genome. Such scaffold maps have already been used with success in other genome projects aiming at getting complete genomes [23, 24].

Once the map was completed, a first series of gaps was closed by assembling the initial set of contigs together with local extensions of contig edges (Fig. 3). Assembly of these extensions benefits from using only a local selection of reads, which usually prevents *de novo* assembly issues associated with duplicated areas of the genome [20]. Moreover, since the size of these extensions was most often close to the insert size of the MP library, many gaps were successfully solved this way. However, in the case of regions with low coverage and/or low complexity or when distance between two successive large contigs was larger than two MP insert size, this strategy only allowed us to partially fill the gap. This additional assembly step led to the generation of supercontigs, the contig composition of which was checked by comparison with the genome map, before being merged into a single, gapped circular genome (Fig. 1).

At this stage, all remaining gaps required a close visual inspection to be solved. Indeed, these gaps resulted either from tricky genome configurations (*e.g.*, repeats at contig edges or in inter-contig regions), or from extremely low sequencing coverage, which may have led the CLC assembler to discard the corresponding reads at an early stage of the assembly. As in the strategy used by the IMAGE software [20], this step was performed using another round of iterative contig extensions but, in our case, extensions were made by retrieving then aligning reads matching a ~20 bp motif at the supercontig edges. This refining step allowed us to get a high quality ungapped genome sequence.

The final smoothing of the genome sequence consisted in the remapping of MPs (with a distance constraint) onto the genome in order to correct for possible coverage anomalies and for unresolved polymorphisms in highly conserved multi-copy genes [25], such as the *psbA* family, coding for the different D1 isoforms of photosystem II [26] that were initially assembled into a single consensus contig. This step also allowed us to get rid of the few residual issues, such as intra-contig chimera generated during the CLC assembly step (identified by coverage drops) or successions of close repeats collapsed into a single consensus sequence (identified by coverage rises; [27, 28]). In the case of WH8103, the sequence of the highly repetitive *swmB* gene [18], was manually reconstructed based on an estimation of the copy numbers for each repetitive motif. At last, this remapping step also allowed us to check that the read coverage was homogeneous along the final version of the genome.

## Conclusions

WiseScaffolder is a novel scaffolder designed not only to produce high quality scaffolds, but also maps of oriented contigs, allowing the user to manually refine and eventually close the genome sequence. Using three datasets representative of both prokaryotic and eukaryotic genomes and of the current standard of the Illumina technology, the automatic part of this pipeline led to a very limited number of scaffolds and was found to perform better than the well-known scaffolders SSPACE and SOPRA and the most recent one SCARPA. The reliability of the scaffolds obtained for *Synechococcus* sp. WH8103 was confirmed by comparison with a closely related reference genome sequenced by Sanger technology [29]. Here, we also propose guidelines for genome finishing that use the outputs generated by WiseScaffolder to refine the scaffolding. In our hands, this pipeline led to the complete closure of the WH8103 genome and was also successfully applied to 31 additional *Synechococcus* genomes, among which 28 were closed and only 1 to 3 gaps remained in the 3 incomplete, yet circularized genomes (http://application.sb-roscoff.fr/cyanorak/). Altogether, our results on both prokaryotes and eukaryotes genomes indicate that the automatic part of this pipeline should perform better than previous scaffolders for most genome projects using MPs, while the semi-automatic finishing part of the pipeline is applicable, with reasonable human cost, only when the assembly step results in a limited number of contigs.

It is worth noting that although the construction of MP libraries remains technically challenging, they constitute an undeniable asset for genome scaffolding and computational finishing and their use is therefore highly recommended for forthcoming projects aiming at getting complete genomes. Upcoming improvements in NGS technologies, including increase of read lengths, accuracy and length of MP insert sizes, which should ideally be larger than 6 kb in order to get over the ribosomal operon, should lead to even better performance of WiseScaffolder. Furthermore, even though the advent of single molecule real time sequencing is expected to further reduce the need for genome assembly, scaffolding and finishing, WiseScaffolder will remain valuable as long as the result of assemblers will not be a single circular chromosome. Finally, we anticipate that WiseScaffolder will also be a key asset to reconstruct uncultured genomes from complex environmental communities using MP read metagenomes, a current challenge in environmental genomics [30].

## Materials and methods

### Genomic material

*Synechococcus* sp. WH8103 (Roscoff Culture Collection strain: RCC2366), a clonal but non-axenic strain, was isolated by J. Waterbury in 1981 in the Northwestern Atlantic Ocean (28° 30' 0" N, 67° 23' 30" W). The genome sequencing dataset consists of two libraries sequenced by Illumina technology: a short insert PE library for genome assembly and a long insert MP library designed for genome scaffolding. Raw datasets are available via the Sequence Read Archive (SRA) under the EBI accession number ERP006796. The PE library was composed of 2 × 2.10^7^ reads of 101 bp with an average insert size of 75 bp and the MP library of 2 × 4.1.10^7^ reads of 101 bp with an average insert size of 4.2 kb (Table 1). In both cases, the coverage was around 800-fold. The closely related complete genome of *Synechococcus* sp. WH8102, previously sequenced using Sanger technology [29], was used as a reference to assess the quality of the scaffolding.

Two other datasets from the GAGE study [14], available at http://gage.cbcb.umd.edu/data/ and for which complete reference sequences are available, were used in this study to test the efficiency of WiseScaffolder with regard to other scaffolders. These include a second prokaryote, *R. sphaeroides,* as well as a representative eukaryotic genome, *Homo sapiens* Chr.14 (Table 1). In the case of *R. sphaeroides*, the dataset consists of an MP library of 2 × 10^6^ reads of 101 bp with a 3,500 bp insert size and contigs generated using the Bambus2 assembler [16]. For *H. sapiens* Chr.14, we used contigs assembled using CABOG (Celera Assembler with the Best Overlap Graph) and an MP library of 2.2 × 10^7^ reads of 101 bp with a 2,280-2,800 bp insert size [14, 17].

### Generating input data for WiseScaffolder

Three input files are necessary to run WiseScaffolder (Fig. 1): i) a series of contigs (multifasta file) generated by *de novo* assembly, ii) a ‘*contig info table*’ containing three columns specifying the contig identifiers as well as coverage and length of each contig, and iii) a ‘*MP mapping table*’ specifying the identifiers of MP reads, their contig best matches and their positions (5′- and 3′-ends) on contigs. A test data set including these three files and a handbook are available at http://abims.sb-roscoff.fr/wisescaffolder.

#### *Generation of* Synechococcus *sp. WH8103 contigs*

Initial assemblies of PE reads were realized *de novo* using the CLC AssemblyCell^©^ 4.10 (CLC Inc, Aarhus, Denmark) with default parameters. In order to select only contigs corresponding to *Synechococcus* (and not to co-occurring heterotrophic bacterial contaminants), PE reads were re-mapped onto contigs using CLC mapper, then average coverage values for each contig were obtained using CLC assembly info. Based on the average coverage of *Synechococcus* contigs, a threshold value of 500 x was used.

#### Mapping of MP onto contigs

This task was realized using either MegaBLAST [31] or Bowtie2 [32] with default parameters. MegaBLAST can generate tabulated text outputs, which are easy to manipulate by bash scripting. However, both pairs are mapped separately and resulting files then need to be joined to build the custom 7-column tabulated file, one of the two formats accepted by WiseScaffolder. Bowtie2 has the advantage to be much faster and parallelizable. By mapping both pairs simultaneously, it can generate a SAM (Sequence Alignment/Map)-formatted file [33], also directly compatible with WiseScaffolder. In both cases, only the most accurate hits were conserved (*i.e.*, full length alignments with 0–3 mismatches).

### Scaffolding steps using WiseScaffolder

WiseScaffolder requires four successive subcommands (Fig. 1), the parameters of which are described in Additional file 1: Table S1.

#### Dumpconfig

**Fig. 4:**
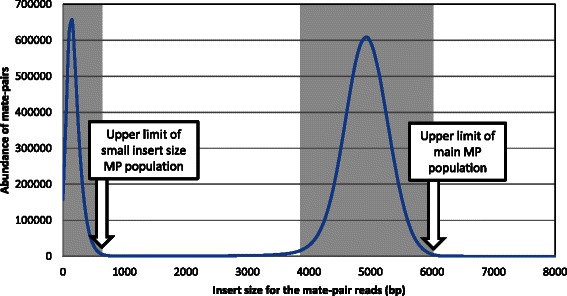
Distribution of the mate-pair insert sizes generated using the WiseScaffolder ‘*preprocess*’ subcommand

This subcommand generates the configuration file, which will be used throughout the scaffolding pipeline. This file specifies several manually editable parameters, namely: i) default names for the main WiseScaffolder outputs, ii) the column indexes that need to be read from both tabulated input files (‘*MP mapping table*’ and ‘*contigs info table*’), iii) the upper limits of insert size distributions for both the main MP and small insert size populations (Fig. 4), the latter needing to be removed from MP links between contigs, iv) a bin size for contig length-based histograms, set by default at 50 bp, and v) the minimal size of big contigs, which is usually set at the upper limit of the insert size of the main MP population. If not known prior to running WiseScaffolder, the latter limits can be visually determined from the ‘*MP insert size graph'* (Fig. 4), which is generated by the WiseScaffolder *'preprocess'* subcommand when used with the --dumpfile option (Additional file 1: Table S1). When the small insert size MP population is absent, the corresponding parameter must be set at 0.

#### Preprocess

The main goal of this subcommand is to detect chimerae located within ‘big’ contigs and to estimate the number of copies of each contig, as deduced from their assembly coverage. As a result, this preprocessing step generates two files: the ‘*chimera resolution file*’ and the ‘*contig copy number file*’, which can be further corrected by hand before being re-imported for the automatic scaffolding step (‘*scaffold’* subcommand). Indeed, the latter step can be greatly affected by the presence of chimerae as well as multi-copy genes, which are often assembled into consensus contigs, the coverage of which is a multiple of that observed for single-copy chromosomal regions.

The initial ‘*chimera resolution file*’ is a 5-column table listing all contig identifiers and positions as well as the results of the automatic chimerae detection step (Additional file 1: Figure S2). In principle, a single-copy contig with a size exceeding the MP insert size should have no more than two other neighboring big contigs in the chromosome. Chimera suspicion warnings are raised when a large contig has a significant number of MP links with more than the expected two neighboring big contigs. These contigs are then tagged as potentially being chimeric in the ‘*chimera resolution file*’. The exact location of each chimera component can be determined by plotting the contig ‘*linkage location graph*’ for the chimera candidate (Additional file 1: Figure S3). The ‘*chimera resolution file*’ may then be manually modified by splitting the chimeric contig into two (or more) components with variable overlaps (Additional file 1: Figure S2).

The ‘*contig copy number file*’ provides an estimation of the number of copies of each contig. Based on the fact that the largest contig is likely to be single-copy, this number is calculated as the rounded ratio of the contig coverage to the coverage of the largest contig, both values being found in the’*contigs info table*’. This information is important since only single-copy contigs are considered for the first step of automatic scaffolding.

**Fig. 5:**
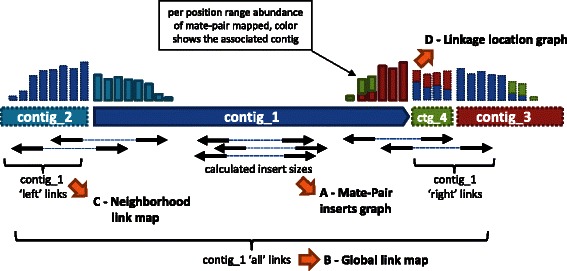
Diagram showing the different outputs generated by the ‘*preprocess*’ subcommand of WiseScaffolfer, called A through D. See text for further details

Additionally, the ‘*preprocess*’ subcommand also generates, using the option --dumpfiles (Additional file 1: Table S1), a number of outputs dedicated to the manual refinement of scaffolding and finishing, as illustrated in Fig. 5. These include i) a ‘*MP insert size graph*’ (see also Fig. 4), showing the distribution of the MP insert sizes, as determined using MP reads mapping on the same contig, ii) a ‘*global link map*’, *i.e.* a symmetric matrix providing for each contig the number of MP reads linking it to other contigs, iii) a ‘*neighborhood link map*’, similar to the ‘*global link map*’ but with an indication of the location of MP reads on the contig (5' or 3' ends) and their orientation with regard to the contig, and iv) a ‘*linkage location graph*’ per contig, used to draw a histogram of the number of links with all other contigs per range of position. Clean versions of both ‘*global link map*’ and ‘*neighborhood link map*’ are also generated after discarding noisy links, *i.e.*, either short-insert MPs or those with an insert size more than 1.5-fold the upper limit of the main MP population (see Fig. 4).

#### Scaffold

This subcommand constitutes the automatic scaffolding step. It uses i) the ‘*chimera resolution file*’ to split chimerae and redistribute their corresponding MP links to the different chimera components, ii) the ‘*contig copy number file*’ that sets the number of copies of each contig, as well as iii) the ‘*MP mapping table*’ and iv) the ‘*contigs info table*’ in order to infer the links between the neighboring contigs, information used to organize contigs into scaffolds. For this purpose, two modules are run successively: the ‘*iterative_scaffold_extender*’ (ISE) and the ‘*small_contig_inserter*’ (SCI).

The ISE module focuses on ordering and orientating ‘big contigs’. The largest contig is used as a seed for a first round of scaffolding. Based on the links between the neighboring contigs, the ISE appends on both sides of the building scaffold, in priority large contigs, otherwise small single-copy contigs, starting with the largest one. This process iterates on both sides of the scaffold until no more big or small single-copy contig can be added. If some large contigs remain unscaffolded, a new scaffold starts with the largest one, and so on until all large contigs are scaffolded. Termination of scaffold extension is generally caused by the presence of multi-copy contigs (*e.g.*, the ribosomal operon), which are excluded by the ISE module to avoid synteny ambiguities due to their occurrence at different locations in the genome.

Subsequently, the SCI module attempts to insert remaining small contigs (single- and multi-copy) within existing scaffolds. For each of these small contigs, their links with neighboring large contigs are analyzed to determine all possible insertion points within the scaffolds and only those congruent with the known succession of big contigs are retained. When multiple copy contigs are inserted at different locations, their copy number is mentioned in the resulting ‘*scaffold maps file*’. It is noteworthy that no small contigs are added at the edges of scaffolds and, as a consequence, no scaffolds are merged by the SCI module.

The ‘*scaffold*’ subcommand eventually generates a ‘*scaffold maps file*’ (see Additional file 1: Figure S4) that can further be manually improved based on the preprocessing outputs.

#### Buildfasta

This subcommand builds the scaffolds as fasta files using the ‘*scaffold maps file*’ and ‘*chimera resolution file*’ as well as the ‘*contigs multifasta file*’ generated prior to scaffolding. Considering that the variability of the insert sizes within the MP library (±1 kb around the peak) does not allow to determine precisely the gap size between contigs and that the quality of the *de novo* assembly makes unlikely the occurrence of large gaps, this subcommand was set to arbitrarily add 50-Ns between contigs.

### Manual improvement of scaffolds and finishing of *Synechococcus* sp. WH8103

#### Completion of the scaffold map

The first manual step after running WiseScaffolder consisted in ordering and orienting the 3 scaffolds to obtain a circular map of the whole chromosome of *Synechococcus* sp. WH8103. To do this, we used the ‘*scaffold maps file*’, which contains the list of contigs within each scaffold, and determined their neighborhoods using two outputs of WiseScaffolder, the ‘*linkage location graphs*’ and the ‘*neighborhood link map*’ (Figs. 1 and 5).

#### *Local reassemblies and* de novo *scaffold assembly*

In order to get the inter-contig sequences, we assembled paired reads complementary to those mapping at contig edges (Fig. 3). These new contigs covering inter-contig areas were assembled together with the initial contigs using Geneious v.R6 (Biomatters; http://www.geneious.com) and the consistency of the resulting supercontigs with the scaffold map was verified in terms of contig order. Additionally, sequence mismatches between new and initial contigs, often resulting from low quality extremities of new contigs, were manually corrected by trimming. The different supercontigs obtained using this strategy were then ordered according to the scaffold map and separated by 50-Ns gaps.

#### Manual gap closure

Remaining gaps were eventually closed by a second type of contig extensions, consisting in extracting then aligning reads (MP or PE) matching a ~20 bp motif at the very edge of the contig to be extended. Read alignments and integration of contig extensions into the gapped genome sequence were both made using Geneious and iterated until complete gap closure.

#### Coverage check

The next step consisted in remapping the MP reads onto the ungapped scaffold in order to determine the read coverage along the assembled genome. Indeed, the sequencing coverage is expected to be rather homogeneous, except in low complexity regions, which display a reduced coverage often due to the presence of homopolymers. Thus, local variations of the coverage might indicate previously undetected errors made during the *de novo* assembly step: coverage drops most often indicate the occurrence of intra-contig chimerae, while highly covered areas are generally observed for collapsed multi-copy genes or repeated domains. In the latter case, the true number of gene or domain copies may be estimated based on the coverage of this specific genomic region with respect to the rest of the genome. It is noteworthy that large, circularizable areas displaying a high coverage might also be due to mobile elements present in several copies with at least one integrated in the chromosome and the other(s) corresponding to independent plasmids.

#### Polymorphism correction

The final step consisted in a global polymorphism correction based on mapping of MP reads onto the assembled genome. First, a weight-position matrix was built, giving at each position the number of A, C, T or G nucleotides found in the reads. The matrix was then analyzed either to correct a nucleotide (when more than 50 % of the reads displayed a different nucleotide than the assembled genome at this position) or to flag the positions when the nucleotide identity was ambiguous (*i.e.*, when two nucleotides were equally abundant, no nucleotides had a frequency higher than 50 % or when the coverage at this position was too low). The script used to perform this step is available on the WiseScaffolder website.

#### Genome finalization

The base numbering of the circularized sequence of *Synechococcus* sp. WH8103 was started 174 bp before *dnaN*, corresponding approximately to the origin of replication. It was then automatically annotated through the Manatee pipeline (Institute of Genome Sciences, Maryland, USA) and submitted to EBI under the accession number LN847356.1.

### Performance and comparison with other scaffolders

The results obtained with WiseScaffolder were compared to that obtained with SSPACE (v2), SOPRA (v1.4.6) and SCARPA (v0.241). While the mapping of the MP library against the contigs is included in the scaffolding process of SSPACE, this step was performed using Bowtie2 [32] with default parameters for SOPRA, SCARPA and WiseScaffolder. For all three datasets, insert sizes used as setting for scaffolding are indicated in Table 1. Additional file 1: Table S2 details the wall-clock running times and memory usage for the four tested scaffolders. Although WiseScaffolder tends to be slightly more memory and time consuming than its counterparts, the memory requirement remains perfectly compatible with current standard cluster capacity and the running time is only a matter of a few hours. Additionally, the Galaxy implementation publicly available at http://webtools.sb-roscoff.fr is associated with a cluster with 256 Gb of memory. Additional file 1: Figure S5 shows the evolution of memory usage and running time for the most demanding ‘*preprocess’* subcommand applied to the 42 contigs of WH8103 for an increasing number of MP mappings. Altogether, these results emphasize the increasing amount of memory required as a function of both the numbers of contigs and mapping results imported during the ‘*preprocess’* and ‘*scaffold’* subcommands. As a consequence, it is recommended to run the application on a cluster with a fairly large amount of memory (~100 Gb). However, WiseScaffolder can also be run efficiently on a personal computer with a limited amount of RAM, with little impact on the resulting assembly, using a subset of MPs that map the different contigs with high accuracy (*i.e.*, full length alignments with less than 4 mismatches). A script allowing to sub-sample MP mapping from a SAM file is available on the WiseScaffolder website.

### Implementation and availability

Developed in Python with a few BioPython dependencies (essentially for the ‘*buildfasta’* subcommand), WiseScaffolder is multi-platform and single-threaded. The code structure potentially allows the addition of scaffolding modules to the chain of actions. WiseScaffolder is open source and was installed on the local Galaxy instance of the ABiMS platform at http://webtools.sb-roscoff.fr. The application, the Galaxy wrapper, a handbook, test files and various peripheral scripts are available at http://abims.sb-roscoff.fr/wisescaffolder.

## Additional file

Additional file 1: Table S1. Parameters to be used with the different WiseScaffolder subcommands. **Table S2.** Wall-clock running times and memory usage for the four scaffolders used in this study. **Figure S1.** Whole genome alignments of *Synechococcus *spp. WH8103 and WH8102. **Figure S2.** Example of a ‘*chimerare solution file’* before and after resolution of a chimeric contig. **Figure S3.** Schematic representation of the linkage location graph for the chimeric contig_1, consisting in two chimera components sharing a common sequence. **Figure S4.** Description of the scaffolds as generated by the *scaffold* subcommand and after reconstruction of the genome map. **Figure S5.** Performances of WiseScaffolder *preprocess *subcommand with *Synechococcus* sp. WH8103. (PDF 587 kb)

## Abbreviations

NGS: Next Generation Sequencing
MP: mate-pairs
PE: pair-ends
ISE: iterative scaffold extender
SCE: small contig inserter
