# Supplementary material for: WiseScaffolder: an algorithm for the semi-automatic scaffolding of Next Generation Sequencing data
Source: BMC Bioinformatics. 2015 Sep 3;16:281. doi: 10.1186/s12859-015-0705-y (PMC4559175; doi:10.1186/s12859-015-0705-y)
Supplement: Additional file 1: Table S1. — Parameters to be used with the different WiseScaffolder subcommands. Table S2. Wall-clock running times and memory usage for the four scaffolders used in this study. Figure S1. Whole genome alignments of Synechococcus spp. WH8103 and WH8102. Figure S2. Example of a ‘chimerare solution file’ before and after resolution of a chimeric contig. Figure S3. Schematic representation of the linkage location graph for the chimeric contig_1, consisting in two chimera components sharing a common sequence. Figure S4. Description of the scaffolds as generated by the scaffold subcommand and after reconstruction of the genome map. Figure S5. Performances of WiseScaffolder preprocess subcommand with Synechococcus sp. WH8103. (PDF 587 kb) [file 12859_2015_705_MOESM1_ESM.pdf]

Supplementary Table S1: Parameters to be used with the different WiseScaffolder subcommands

Parameters	Short Name	Long Name	SUBCOMMANDS dumpconfig	preprocess	scaffold	buildfasta
Configuration file output name		--configout	X			
Maximal insert size for the mate-pair library	-i	--insertsize	(X)	(X)	(X)	
Minimal length for 'big contigs' (generally equals the insert size)	-b	--bigcontigminimalsize	(X)	(X)	(X)	
Configuration file input name		--configin		X	X	
Contig info table	-c	--contig		X	X	
Mate-pair mapping table	-m	--matepairs		X	X	
Enable generation of manual scaffolding output files		--dumpfiles		(X)		
Chimera resolution file	-k	--kimera		(X)	(X)	(X)
Contig copy number file	-v	--coverage		(X)	(X)	
Scaffold description output file		--scaffoldout			X	
Contigs multifasta	-f	--fastafile				X
Scaffold description input file	-s	--scaffoldin				X
Directory name for the final fasta files of scaffolds and unscaffolded contigs	-r	--resultdir				X
Enable verbose mode	-p	--progress	(X)	(X)	(X)	(X)
Enable debug mode	-d	--debug	(X)	(X)	(X)	(X)
Display the help message, depending on the subcommand	-h	--help	(X)	(X)	(X)	(X)

X: this option is required to run a given subcommand, (X): this option is optionnal and will override a corresponding parameter in the configuration file.

Supplementary Table S2: Wall-clock running times and memory usage for the four scaffolders used in this study.

		WiseScaffolder	SSPACE	SOPRA	SCARPA
***Synechococcus* sp.**	Running time (min)	86[1] + 61[2] + 6[3]	217[4]	33[1] + 4[3]	45[1] + 1[3]
**WH8103**	Max. memory usage (Gb)	48	6	5	12
Rhodobacter	Running time (min)	1[1] + 3[2] + 1[3]	5[4]	0[1] + 20[3]	1[1] + 0[3]
sphaeroides	Max. memory usage (Gb)	3	3	2	3
Homo sapiens	Running time (min)	20[1] + 150[2] + 40[3]	23[4]	7[1] + 714[3]	7[1] + 2[3]
**Chr.14**	Max. memory usage (Gb)	171	6	23	5

[1] Bowtie2 mapping on 25 nodes; [2] WiseScaffolder preprocessing; [3] scaffolder processing; [4] scaffolder processing, including mapping.

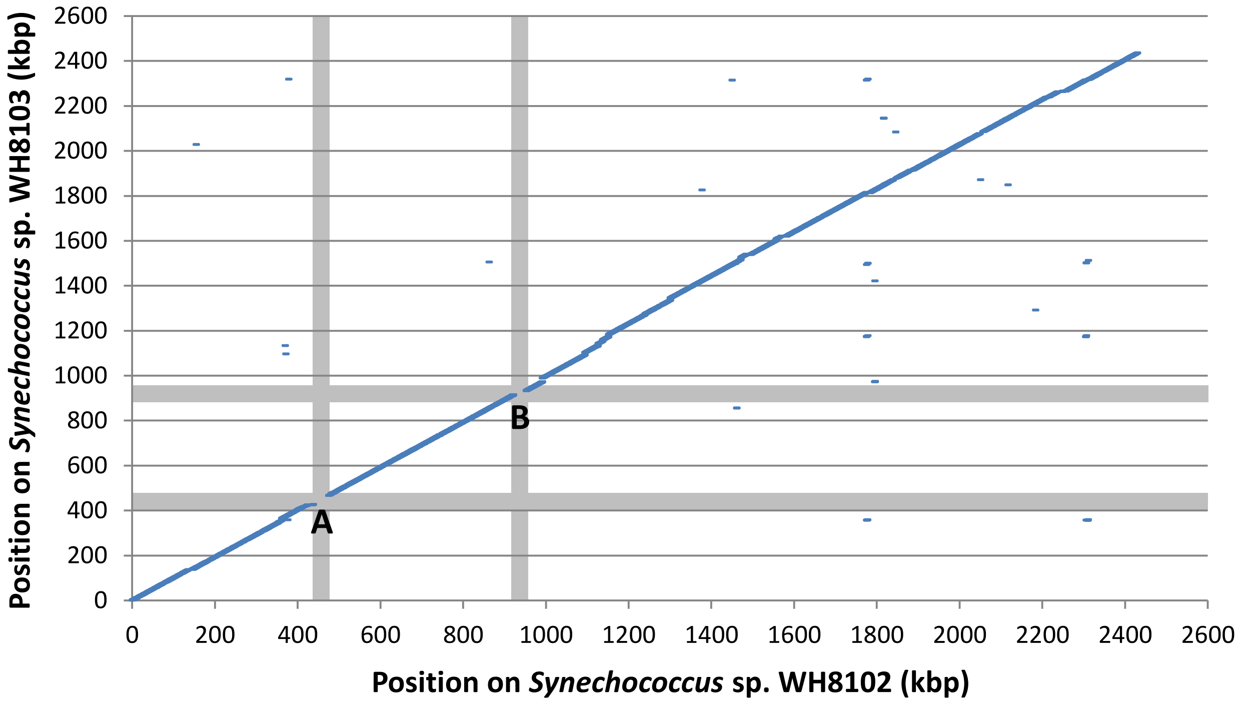

Supplementary Figure S1: Whole genome alignments of *Synechococcus* spp. WH8103 and WH8102 realized using MUMmer (Delcher *et al.*, 2003). Two regions with low homology are indicated by light grey areas: A. large gap around position 450 kbp corresponding to a genomic island with similar size but different gene content in both genomes, B. large gap around position 930 kpb corresponding to a giant ORF encoding the motility-associated gene *swmB* (~32 kb; McCarren and Brahamsha, 2007)

Before chimerae resolution :

chimera_id	contig_id	start	stop	candidate?
contig_1	contig_1	1	125000	True
contig_2	contig_2	1	250000	False
…				

After chimerae resolution:

contig_1A	contig_1	1	**75000**	True
contig_1B	contig_1	**74000**	125000	True
contig_2	contig_2	1	250000	False
…				

Supplementary Figure S2: Example of a '*chimera resolution file'* before and after resolution of a chimeric contig. Contig identifiers are listed in columns 1 and 2, the start and end positions of each contig in columns 3 and 4 and the last column indicates whether a chimera has been detected within the contig. After visual determination of the exact location of the chimera components on the '*linkage location graph*', the line corresponding to the chimeric contig is duplicated and the chimera components are renamed in column 1 with a unique identifier (here contig_1A and contig_1B). The initial contig identifier (column 2) remains unchanged to specify which chimeric contig needs to be split. Columns 3 and 4 describe the positions of the resulting components after chimera resolution. In the example shown, the initial 125 kbp contig_1 was split into two contigs of 75 kbp and 51 kbp (with a 1 kbp duplicated region) that can then be scaffolded independently.

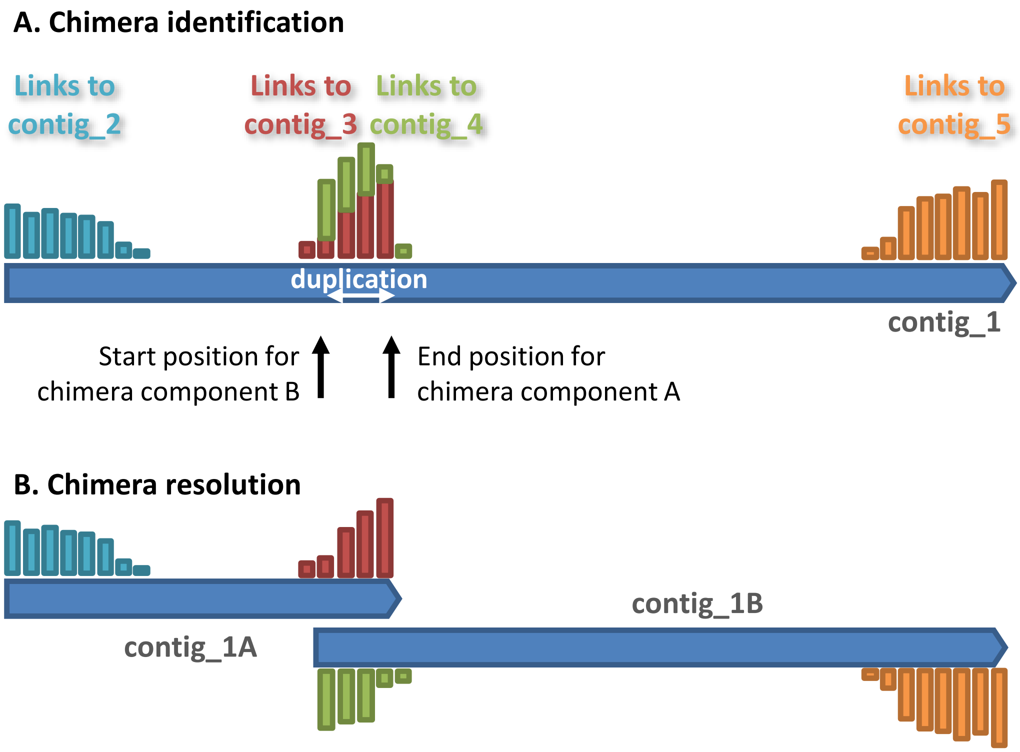

Supplementary Figure S3: Schematic representation of the linkage location graph for the chimeric contig_1, consisting in two chimera components sharing a common sequence. (A), before chimera resolution, the chimera position is identified by the presence of links between an area located within contig_1 and external contigs (contig_3 and 4); (B), after chimera resolution, the area indicated by a double-headed arrow is manually duplicated to generate two new contigs (contig_1A and 1B).

A

```
[scaffolds]
scaffold_001 - linear: <<+CONTIG_8>> +contig_4 -CONTIG_29 +contig_18
-CONTIG_9 +contig_70 -CONTIG_23 +contig_56[1] +CONTIG_6
scaffold_002 - linear: +CONTIG_3 -contig_78 -contig_38 +CONTIG_1
-contig_50[1] +CONTIG_16 <<+CONTIG_7>> -contig_85[1] +CONTIG_31 +CONTIG_13
-contig_60[1] -CONTIG_40 -contig_60[2] +CONTIG_11 -contig_85[2] -CONTIG_17
+contig_84 +CONTIG_21 +CONTIG_5 -contig_50[2] +CONTIG_2
scaffold_003 - linear: -CONTIG_26 +contig_56[2] <<+CONTIG_12>>
[remaining]
?contig_46; ?contig_47; ?contig_48; ?contig_79; ?contig_71; ?contig_117;
?contig_39; ?contig_36; ?contig_58; ?contig_30; ?contig_82; ?contig_83;
?contig_109
```

B

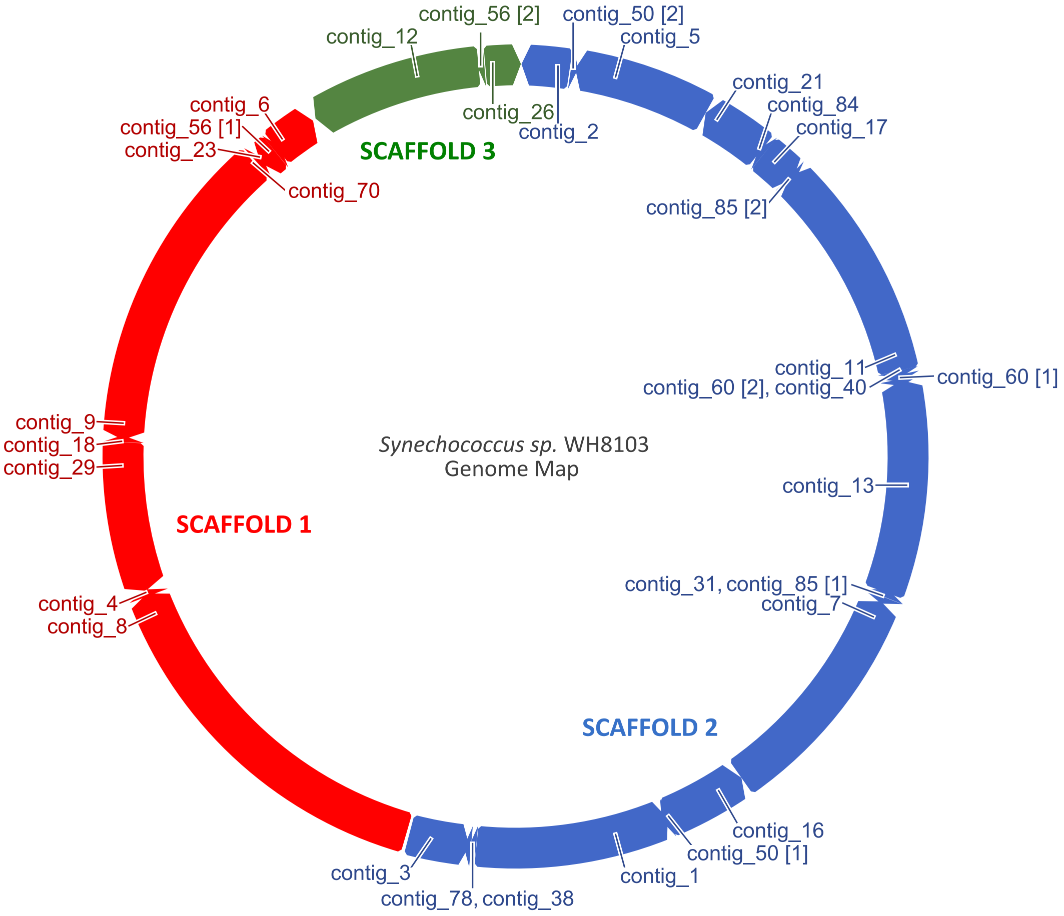

Supplementary Figure S4: (A) Example of the scaffold description file as generated by the scaffold subcommand of WiseScaffolder. A first block describes scaffolds: the largest yet unscaffolded contig used as a seed for the iterative_scaffold_extender (ISE) module is surrounded by double quotes, contigs in capital letters correspond big contigs, and the orientation of contigs within scaffolds is indicated using "+" or "-" for a forward or a reverse orientation, respectively. When small contigs are inserted as multiple copies, the copy number is indicated between square brackets. The second block corresponds to unscaffolded contigs, some of which may be contaminants or simply dismissed multicopy contigs that cannot be integrated in the reconstructed chromosome without ambiguities. (B) Graphical representation of the three scaffolds map after reconstruction of the genome map.

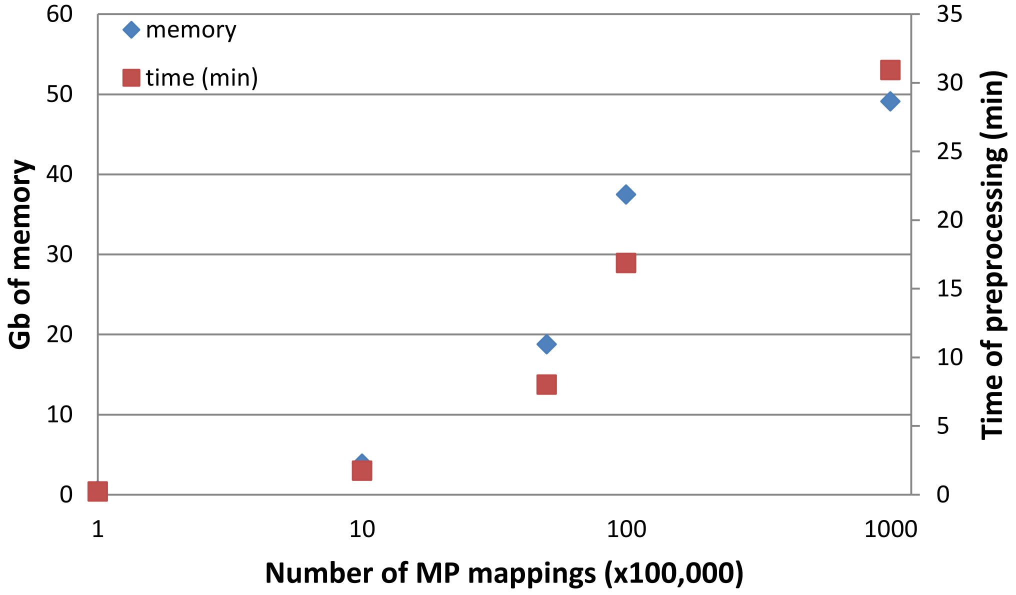

Supplementary Figure S5 : Performances of WiseScaffolder *preprocess* subcommand with *Synechococcus sp.* WH8103
